# Health care systems in Sweden and China: Legal and formal organisational aspects

**DOI:** 10.1186/1478-4505-8-20

**Published:** 2010-06-22

**Authors:** Björn Albin, Katarina Hjelm, Wen Chang Zhang

**Affiliations:** 1 School of Health Sciences and Social Work, Växjö University, and Department of Health Sciences, Division of Geriatric Medicine, Lund University, Sweden; 2 School of Public Health, Fujian Medical University, PR China

## Abstract

**Background:**

Sharing knowledge and experience internationally can provide valuable information, and comparative research can make an important contribution to knowledge about health care and cost-effective use of resources. Descriptions of the organisation of health care in different countries can be found, but no studies have specifically compared the legal and formal organisational systems in Sweden and China.

**Aim:**

To describe and compare health care in Sweden and China with regard to legislation, organisation, and finance.

**Methods:**

Literature reviews were carried out in Sweden and China to identify literature published from 1985 to 2008 using the same keywords. References in recent studies were scrutinized, national legislation and regulations and government reports were searched, and textbooks were searched manually.

**Results:**

The health care systems in Sweden and China show dissimilarities in legislation, organisation, and finance. In Sweden there is one national law concerning health care while in China the law includes the "Hygienic Common Law" and the "Fundamental Health Law" which is under development. There is a tendency towards market-orientated solutions in both countries. Sweden has a well-developed primary health care system while the primary health care system in China is still under development and relies predominantly on hospital-based care concentrated in cities.

**Conclusion:**

Despite dissimilarities in health care systems, Sweden and China have similar basic assumptions, i.e. to combine managerial-organisational efficiency with the humanitarian-egalitarian goals of health care, and both strive to provide better care for all.

## Introduction

### Globalisation and cooperation in health care research and development

To improve health and health care worldwide, international cooperation should include aspects such as attitudes to patients and the organisation of health care services [1]. The increasing number of elderly people will put pressure on health care systems in many countries [2]. Sharing knowledge and experience can give valuable information on successful systems, while comparative analysis and experiences can strengthen international collaboration [3]. Comparative research can make an important contribution to knowledge concerning health care [4], not least in cost-effective use of resources. Western European countries have seen a move towards evidence-based health care to improve the utilisation of resources and ensure high-quality health care, and to upgrade staff competence and medical practices [5].

There are studies describing the organisation of health care in many countries such as Sweden and China, but our literature search indicated that no research has so far specifically compared the legal and formal organisational systems in these two countries although there are general worldwide statistics produced by e.g. WHO and OECD.

Swedish health care is part of the national welfare model, with its roots in the depression of the 1920s and 1930s, when visions of national welfare policies, comprehensive basic financial security, and the right of the entire population to social services on equal terms were conceived. These concepts were developed during an almost unbroken period of Social Democratic government up to the middle of the 1970s [6]. The Swedish health care system has undergone a number of reforms and changes but, in general, the basic structure of the system has been relatively stable. The major reforms since 1970 have been due to demographic changes, with an increasing number of elderly people and people with complex non-communicable diseases, and as well as reductions in economic growth, with stagnating industrial development [7].

One of the main changes in health care and social services came in 1992 when responsibility for elderly care was transferred from county councils to local authorities [8]. The aim was to care for elderly persons as far as possible in their own homes or in settings within primary health care. Over time this also led to an increasing number of private actors providing elderly care. Since 1995 the municipalities has also taken over the responsibility for persons with long-term mental illness and all kinds of disabilities. This includes responsibilities for living conditions, employment and daily support [9]. Another recent change is the ongoing reform to freely choose primary health care centre based on the patients desire [10].

During the past 20 years China has undergone rapid change with a transition from a planned socialist economy to an economy with more market influences [11], and thus a growing non government health care sector [12] often in terms of small practices outside the hospital [13]. The Chinese government has made economic development a top priority, at the expense of public health, especially in terms of access to health care for the 800 million people living in rural areas [14]. The major changes are the growing inequality in access to health care, increasing costs of medical care, and the deterioration of preventive programmes in some areas [15]. There is a need to reorganise the public health system by strengthening connections between the various public health organisations [14]. The Chinese government has increasingly recognised the importance of investing in health, and improving health care services has become a key element in economic development plans. China is now facing problems that have been evident in Sweden for some time. With increasing economic growth, changes in lifestyle towards that in Western cultures are now influencing the pattern of health and diseases [16]. China also has a large elderly population, and the policy of one child per family also has special implications for elderly care.

It is thus of interest to compare the influences on the organisation of health care of a rapidly changing society, as in China, with Sweden and its relatively unchanged structure despite societal changes. Other countries can learn from the comparisons between different countries by taking a systematic approach to the formulation and implementation of health care reforms [15].

## Aim

To describe and compare health care in Sweden and China with regard to legislation, organisation, and finance.

## Method

Literature reviews were carried out by University librarians and the authors in Sweden and China. In Sweden the PUBMED, CINAHL, LIBRIS and ELIN databases were searched, identifying literature, in Swedish and English, published from 1985 to 2008. In China, the following databases were searched, identifying literature published, in Chinese and English, during the same period: VIP Chinese Science and Technology periodical, Chinese periodical net, the Wanfang database, the website of the Chinese Ministry of Health http://www.moh.gov.cn and the Health Economics Institute website http://www.nhei.cn/. The People's Medical Publishing House series of textbooks on health management (e.g. Health Economics, Social Medicine) was also consulted.

The main keywords used in both countries were: China, Sweden, Health care organisation, Health care systems, Health/disease patterns, Patients' rights/responsibilities, and Financing of health care. References in recent studies were scrutinised, national legislation and regulations and government reports were searched, and textbooks were searched manually. Reference lists of obtained articles were also hand searched. Discussions were held with front-line researchers regarding key-references. Older material was included when it was connected to later research. The knowledge base relevant to the organisation of health care in Sweden and China was reviewed systematically. Solely abstract and unpublished studies were excluded from the study.

## Results

### Legislation

#### Laws regulating health care in Sweden

Health care in Sweden is governed by laws, ordinances, and regulations. Laws are instituted by parliament, ordinances are developed by the government, and regulations are drawn up by public authorities such as the National Board of Health and Welfare.

The framework for the regulation of all health care services in Sweden is the Swedish Health and Medical Services Act [17], which states the overall objective of health and medical care: *"Good health and care for the whole population on equal terms"*. The Act also states that health care shall be of a high standard and satisfy the patient's need for security, be easily accessible, be based on respect for the patient's right to self-determination and integrity, and promote good communication between the patient and health care personnel.

More detailed enactments and regulations are to be found in Svensk Författningssamling and regulations from e.g. the National Board of Health and Welfare. Each provider of health care may also develop guidelines according to national legislation, ordinances, and regulations.

#### Laws regulating health care in China

Chinese health care is governed by the legal document formulated by the National People's Congress and its Standing Committee. Health care legislation is divided into two parts, one instituted by the National People's Congress, the other by the Standing Committee of the National People's Congress. The first, called the Fundamental Health Law, has not yet been completed. The other part is the hygienic common law [18].

Chinese health care is regulated by the normative legal document formulated by the State Department. The State Department has hitherto constituted 32 health care regulations. Department rules are those legally constituted by the administration of public health, such as the State Food and Drug Administration, the Family Planning Commission of China, Entry-Exit Inspection and the Quarantine Bureau, etc. In total, there are 92 department rules in the area of health care [18].

In 1997, the Chinese Communist Party's Central Committee and the State Council on the Reform and Development of the Health Care System Reform and Development has defined the main goal of health care reform in China thus: *"to set up, by 2010, a health system adapted to the new economic system such that the main indices of public health in developed areas approach or reach those of a middle-income country, and those in developing areas reach the highest levels in developing countries."*

China emphasizes rational use of health resources and improvement of health status [9]The concrete goals include: enhancing public health by improving the quality and efficiency of health services; providing basic medical insurance for all workers in urban areas; using existing resources efficiently; developing community health services; developing and perfecting rural cooperative medical care; strengthening the development of rural health organisations; strengthening areas such as the prevention of disease and promoting public health [18].

### The patients' role in Swedish health care expressed in the legislation

According to the Swedish Health and Medical Services Act of 1982, the emphasis in health care is on a humane and understanding attitude towards each individual. A person seeking health care has the right to dignity and integrity. Patients must be given personal information concerning their health and the methods of treatment available. If this information cannot be given to the patient, it is supplied to a close relative. Care and treatment shall, as far as possible, be designed and carried out in consultation with the patient.

### The patients' role in Chinese health care expressed in the legislation

The patients' role is not clearly defined in the Chinese legislation but it is claimed that it aims on protecting the patient's rights and independence [19]. However, at present no special law defines how patients' rights are to be protected, only some prescripts are found in the health laws and regulations currently in force:

• the reasonable and limited right of independent choice of medical treatment,

• the right to be informed about their illness, and the right to consent to medical treatment,

• the right to monitor medical services and protect patients' rights and interests.

• cooperating with the doctor during diagnosis and treatment,

• paying for medical services,

• observing regulations prescribed by the hospital during treatment [19].

### Health care staff's role in Swedish health care expressed in the legislation

When alternative forms of treatment which are in accord with science and proven experience are available, the county council shall allow the patient to choose the one preferred [17]. The county council shall provide the patient with the chosen treatment if this is deemed justifiable by the illness/injury and the cost of treatment.

The county council shall give a patient with life-threatening or particularly serious illness/injury the possibility of obtaining, within his or her own county council area or outside it, a renewed medical assessment if science and proven experience does not provide unambiguous guidance, and if a medical decision may entail special risks for the patient, or is of great importance for the patient's future quality of life. The patient shall be offered the treatment the renewed assessment may occasion [17].

During the 1990s, quality assurance became more important in health care [20]. Areas in focus were the availability of health services and client focus, and the degree to which they are knowledge-based. A number of regulations on quality issues were issued by the National Board of Health and Welfare in 1994.

### Health care staff's role in Chinese health care expressed in the legislation

In China, health care staff protect the people's health. In 1977, Engel pointed out that, "It is inevitable that the direction of medical development would gradually turn from the simple biomedical model into bio-psycho-social medical model" [21]. This concept was first introduced into China in the National Medical Dialectic Conference in 1981. After this, the attitude to the patient was supposed to change. The focus was to switch from therapy to prevention, from physical care to psychological care, from inpatient to outpatient care, and from technical service to more social service, to enhance the level of prevention, health care, and rehabilitation [22].

### Organisation

#### Different levels of health care and specialisation in Sweden

The Swedish health system has three administrative and political levels: The national level, the county level, and the municipality level. These are all represented by directly elected political bodies, and all have the right to finance their activities by levying taxes and fees [20].

The national level has the responsibility for law, regulations, monitoring, evaluation, and some national state obligations like national disease control, drug registration and drug control. The county level has the responsibility for all health care services including their public financing. Also, most county councils are the major providers of health services in their councils. Swedish health care is largely financed by a county council tax paid on individual incomes. The municipality level is in charge of care for the elderly, including basic health care in the homes of the elderly or in nursing homes.

The most important role of the Swedish government is to establish basic principles for health care through laws and ordinances, such as the Health and Medical Services Act of 1982 instead of detailed regulations [20]. However, the central government has in contrast to county councils no responsibility for service provision. The main responsibility within the national government lies within the Ministry of Health and Social Affairs, which draws up terms of reference for government commissions, drafts proposals for parliament on new legislation, and prepares other government regulations. The National Board of Health and Welfare acts as the government's central advisory and supervisory agency with the task of following up and evaluating the services provided and developing national guidelines for good medical practice.

The Swedish health system is functionally divided into three levels: Primary health care, county hospital(s) and regional hospitals with varying degrees of specialisation, which together form a chain of health care in which each link or level has its own area of responsibility [23]. The aim of this system is to provide health care at the right level with the most efficient utilisation of resources.

*Primary health care *(PHC) constitutes the basis of the Swedish health care system. It is responsible for public health and the treatment of diseases and injuries not requiring hospitalisation or specialist care. PHC is organised into health care centres, with outpatient clinics staffed by GPs, nurses, district nurses and nursing assistants serving people living in defined geographical areas. Primary health care is responsible for the health of a certain population, as well as offering health care for the individual based on a holistic perspective [23].

At PHC centres district nurses often run outpatient care facilities and sometimes also visit immobilised patients who are cared for in their homes. Home nursing services are often provided by nursing homes or health care centres, mainly staffed by groups of nursing auxiliaries led by a district nurse or a general nurse. In some cases with high demands on staff competence, only district nurses or nurses provide this type of care. Staffing of nursing homes is similar to that of the home nursing service, but the staff are often supervised by a nurse or, in some cases, a district nurse [24].

*County level *is the second level comprising county hospitals and district hospitals. Patients needing more specialised care can be referred from PHC to hospitals offering care in a number of specialist fields, e.g. dermatology, ophthalmology, ear, nose and throat, etc.

*The regional level *is the third level of health care comprising regional and university hospitals where the most highly specialised care is provided, e.g. coronary surgery, organ transplants, etc. They are also centres of medical and scientific research and teaching.

Because of the significant reduction in the budget, the trend in Swedish health care is to move patients out of expensive beds and into less expensive forms of care, e.g. PHC or home care [8]. Health care is mainly provided by public authorities, and private care is still rare [25].

#### Different levels of health care and specialisation in China

In China the governments responsibility for provision and financing has changed and local government institutions and individual households have became more important [26]. Regional health care planning providers try to satisfy requirements from the national government by setting guidelines for the allocation of the health care resources across the provinces. There are examples of provinces that have implemented different monitoring systems and control frameworks [27].

China has established a health care system, including health service and medical insurance, that has spread all over the cities and countryside. Health care includes medical service, the Centre for Disease Prevention and Control (CDC), and maternal and paediatric health care. The medical insurance system includes social medical insurance, private medical insurance and medical assistance [28]. However, a recent publication describes a change of the system into four parts: basic medical insurance scheme, urban-resident scheme, rural cooperative medical system, and medical assistance programme [29]. This system is still evolving and there are large variations in what services are provided or the costs in different parts of China.

There are different levels of medical service in China, from village to township to county to prefectural and to provincial and above level [9]. Medical service in rural areas is a kind of social welfare organisation. It is further divided into three sub-levels according to its technical and service level [30].

• *The first level of medical organisations *are mainly established in small cities and towns or in the countryside, e.g. town hospitals, health centres in the city, regional hospitals and the hospitals are run by mines and/or other enterprises.

• *The second level of medical organisations *are mainly the borough hospitals in the county, city or municipality directly under central government. They offer medical services over several communities and they constitute regional preventive technical centres, which form the centre of the three-level system.

• *The third level of medical service organisations *are mainly the large hospitals of the provinces and big cities, and hospitals affiliated to medical colleges. They constitute the technical centres of medical and scientific research and teaching, which form the top of the three-level system [30].

Besides these organisational levels, there are two other important structures in health care, the Centre for Disease Prevention and Control and the Maternal and Children's Health Service.

The Chinese Ministry of Health issued several regulations in 2004 to promote the establishment of a disease prevention and control system. The aim is to improve measures to prevent and control disease and the ability to deal with public health problems, to safeguard the health and safety of the population, and to promote social stability and economic development. This disease prevention and control system is divided into four levels: national, provincial, city, and county level.

The disease prevention and control agencies are organised according to the administrative districts in every city. Each centre is responsible for preventing and controlling disease, health education and promotion, the application of research results and guidelines, technical management and the services within its district [31].

The Maternal and Children's Health Service offers technical guidance for maternal and children's health care in local areas. They have the same status as medical service organisations and the centres for disease prevention and control, and form an important part of the Chinese health service. There is a relatively complete service network for maternal and children's health care, and in 2006 there were 3,021 maternal and children's health service organisations in the country. Chinese maternal and children's health service organisations have been established in provinces, autonomous regions, cities, and municipalities. Maternal and children's health care clinics have been established in urban regions and counties [32].

### Finance

#### Economic aspects of health care and medical insurance in Sweden

In Sweden health care services are mainly funded from taxation, both direct taxation of income and indirect tax on products, services, and employers [33].

Health care services are not free, but patient fees cover only a small part of the total cost of medical care; the rest is covered by the county councils, each of which can set its own patient fees. A ceiling for the individual's expenditure over a year is determined by the central level government [34]. Normally, a visit to a GP at a PHC centre costs 150-200 SEK, and a visit to a hospital costs 250-300 SEK. The charge for hospital stays is regulated by law to no more than 80 SEK per day. If a person has a health problem she/he should first visit a GP at the PHC centre, and the GP will decide if referral to a hospital or more specialised care is necessary [20].

National guidelines have been developed concerning the priority of county councils' health care to control the cost of health care and to make sure the available resources are used as efficiently as possible [35].

#### Economic aspects of health care and medical insurance in China

Payment for health care services in China is mainly based on out-of-pocket payment [9]. One of the major problems has been the increasing costs for persons using health care services [29]. Because of the imbalance in economic development in China, it is impossible to establish a uniform national system for medical insurance. Thus, the medical insurance system covers mainly social medical insurance, occupational medical insurance, and medical assistance. The construction of a medical insurance system in China is now being initiated [36]. Examples of the systems introduced are the New Cooperative Medical Schemes(NCMS), urban staff medical insurance, urban residents medical insurance and medical assistance and private medical insurance [9,29]. Chinese social medical insurance can be divided into basic medical insurance, supplementary medical insurance, and private medical insurance.

*Basic medical insurance *is the main, elementary kind of medical insurance in China. In 1993, it was decided to establish a basic medical insurance system throughout the country that is adapted to the productivity level of the primary stage of socialism, meets the needs of the socialist market economy, sufficiently takes into account the endurance capacity of finance, enterprise, and the individual, and guarantees the basic medical health of workers. This is the ideology guiding the reform of the Chinese urban medical health system. According to the governmental decision, three principles should be adhered to in the process of establishing basic medical insurance for urban workers. Firstly, all the urban enterprises and their workers must participate in basic medical insurance. Secondly, the basic premium is to be paid by both the employer and the employees. Thirdly, the basic medical insurance fund comes from both the social integrated fund and individual discount [37].

*Supplementary medical insurance *in China is made available through workplaces. This insurance is established through national regulations and the government's standard instruction. Enterprises are responsible for funding this system, which is a supplement to basic medical insurance [37].

The traditional health care system in China consists mainly of medical care through government and institutional agencies, labour insurance medical care through the state-owned and collective enterprises in urban areas, and cooperative medical care in rural areas. A new medical insurance system is now being established, which provides broad scope for the development of private medical insurance. *Private medical insurance *is attracting increasing attention from the general population, and is becoming an important constituent in the medical insurance system. The government is creating the conditions necessary to promote private medical insurance, for example, by encouraging people to voluntarily join private medical insurance schemes. Thus, every large Chinese commercial insurance company realises the huge scope for development in the commercial medical insurance sector [14].

The Chinese medical assistance system is a social security system in which the nation and society provide assistance to those who cannot obtain basic medical services, and its main form is low-level free medical care. At the end of June, 2003, only 10 million urban workers in China had medical insurance, accounting for roughly 20% of the urban population. Only 10% of workers in rural areas had access to the new rural cooperative medical care, and only 3% of the population had commercial private medical insurance. The medical assistance system guarantees an individual fundamental medical aid, and it is "the final defence line" of social medical security [38].

## Discussion and Conclusions

This article compares the health care systems in Sweden and China with regard to legislation, organisation, and finance. The comparison shows that two different health care systems have been developed in the countries studied. Sweden has clear national legislation concerning health care [17], while China currently has a health care law consisting of two different parts [18], one of which has not been enacted. The main goals stated so far differ from those of Swedish legislation. While Swedish legislation focuses on "good health and care for the whole population on equal terms", Chinese legislation focuses on the development of the health care organisation and accepts differences within the country in the degree of development. Sweden has a system of well-developed primary health care and in-hospital care available at a general or a highly specialised level [39], while the health care system in China is still under development and relies predominantly on hospital-based care concentrated in cities [40]. This is further underpinned by the fact that a proposal has been made to establish a special medical care system for rural areas [41]. In Sweden, most of the responsibility for health care is decentralised to the county councils [6], in contrast to China where central government has the main responsibility [42].

Organisations within the Chinese health service are divided into different levels to allow them to run better, but this also introduces inequality. Many problems beset the Chinese health service: the irrational collocation of health resources, unfair privileges, the delay in implementing health measures in the countryside, and lack of government commitment to health. The development of basic health service organisations faces unprecedented challenges [43]. In some towns, health personnel are only paid the basic wage, and sometimes this cannot be paid on time, which reduces the enthusiasm of health workers.

Regarding health care in China, the goal is to develop a system adapted to a new economic system [44], while the situation in Sweden has been relatively unchanged for a long period due to few changes in the economic system and political governance [6]. In both countries there is a tendency towards private initiatives (market-orientated solutions) in health care as well as in society [39,45]. In Sweden, the change started in the 1990s as a consequence of the recession and, for the first time during a long period, a strained health care budget [39]. A measure of the level of development of a society is its health care budget [2]. In China the total health expenditure is still increasing, from 74.4 billion RMB in 1990, 502.6 in 2001 to 759.0 billion RMB in 2004 [46], while the cost in Sweden has decreased and stabilised since the latter half of the 1990s. Thus, China should still be considered a country under development.

In Sweden, patients are protected against the high costs of health care and loss of earnings due to illness by the national insurance system [20,34]. China has still not established a national medical insurance system. The Proposal on Establishing a New Rural Cooperative Medical Care System (2003) states that the new rural cooperative medical care system should be introduced and fully operational in 2010. The target is to cover the whole nation and to alleviate the financial burden on farmers caused by disease, and to improve the general health of the rural population [36].

At present, many workers in state-owned enterprises are reluctant to take jobs in private enterprises, not only because of their mentality concerning employment, but also because social security does not cover these enterprises, which leads to worries about their future security [46]. Therefore, the establishment of a medical insurance system covering all enterprises and employees in urban areas will be beneficial in changing workers' conceptions of employment, opening up more avenues for employment, and accelerating the reform of state-owned enterprises.

The Swedish Health and Medical Service Act gives patients many rights and opportunities to influence their care through consultation with health care staff, while Chinese health legislation focuses on the principle of maintaining patients' independence but does not have a special law to protect patients' rights. Chinese patients have limited rights, for example, in the choice of treatment, but the law also states obligations of the patients, e.g. cooperation with the doctor in diagnosis and treatment, paying for medical treatment, observing regulations prescribed by hospitals, etc. Thus, the attitude of health care staff to patients in Sweden is based on a humane and understanding attitude towards each individual [17], while in China the emphasis is on maintaining the patient's independence, and changing the concept of the health service from treatment to prevention [41]. In both countries the focus has changed from a biomedical to a bio-psycho-social model, with a more holistic view of the individual, and from treatment inside to outside the hospital [42]. However, in Sweden the development of quality assurance has progressed further as a result of the higher developmental level.

The results of a literature review depend on the possibility of finding the available information. Both countries studied are technically well developed and thus existing databases should guarantee that the relevant literature can be found. One strength of this study is that the same keywords were used in the literature search. On the other hand, a difficulty is that the interpretation of keywords may be influenced by the cultural values and the attitudes of the researchers. Another difficulty and a limitation of the study is to comprehend and get access to relevant information about the health care system in such a big a country as China with rapid changes and varying conditions in different regions/provinces. However, an international comparison by scientists with different cultural backgrounds is a strength in that it opens up different perspectives that may be complementary. In the course of this work discussions have taken place by e-mail and personal meetings between the researchers. Sharing knowledge, resources, and skills has the power to achieve optimal outcomes more easily than trying to accomplish such objectives in isolation [3]. Reflections by comparing and contrasting different health care systems with international colleagues might lead to questioning everyday practice.

In conclusion, the studied health care systems showed dissimilarities in legislation, organisation, and finance, but the two systems are based on similar basic assumptions, i.e., combining managerial-organisational efficiency with the humanitarian-egalitarian goals of health care, and both of them are continuously striving to provide better health care for all but Swedish health-care services are well organised and well financed. Through international cooperation, we can find new approaches in the field of health care, and appropriate modern measures should be adopted in order to achieve greater economies and more effective programmes.

## Competing interests

The authors declare they have no competing interest.

## Authors' contributions

The literature reviews were made by BA and KH in Sweden and by WZ in China. The first draft of this article was composed by BA and KA and revised by all authors. All authors has approved of the final version of the manuscript.

## References

[B1] Frenk J Gómes-Dantés O Globalisation and the Challenges to Health Systems British Medical Journal 2002 325 95 97 10.1136/bmj.325.7355.95 12114243 PMC1123638

[B2] WHO World Health Organization. Health Systems: Improving Performance 2000 Geneva: The World Health Report

[B3] Rolfe M Bryar R Hjelm K Apelqvist J Fletcher M Anderson BL International collaboration to address common problems in health care: Processes, practicalities and power International Nursing Review 2004 51 140 148 10.1111/j.1466-7657.2004.00237.x 15285740

[B4] Ellefsen B Health visiting in Scotland and Norway: Commonalities and differences Public Health Nursing 2001 18 318 26 10.1046/j.1525-1446.2001.00318.x 11559415

[B5] Marchevsky AM Wick MR Evidence-based medicine, medical decision analysis, and pathology Human Pathology 2004 35 1179 1188 10.1016/j.humpath.2004.06.004 15492984

[B6] Socialstyrelsen Health care and social services in seven European countries 1993 Stockholm: Socialstyrelsen

[B7] Socialstyrelsen Folkhälsorapport (Public Health Report) 2006 Stockholm: Socialstyrelsen

[B8] Socialstyrelsen Nationell handlingsplan för äldrepolitiken - Lägesrapport 2001 (National plan of action for geriatric policy - Progress report for 2001) 2001 Stockholm: Socialstyrelsen

[B9] Dong HJ Health financing systems & drug use in rural China 2000 Stockholm: Department of Public Health Sciences, Karolinska Institutet, Stockholm, Sweden

[B10] Burström B Market-oriented, demand-driven health care reforms and equity in health and health care utilization in Sweden Int J Health Serv 2009 39 2 271 85 10.2190/HS.39.2.c 19492625

[B11] Shanlian Hu Health Economics 2003 Shanghai: Fudan University Publishing House 3

[B12] Liu Y Development of the rural health insurance system in China Health Policy and Planning 2004 19 3 159 65 10.1093/heapol/czh019 15070864

[B13] Liu Y Berman P Yip W Liang H Meng Q Qu J Li Z Health care in China: The role of non-government providers Health Policy 2006 77 212 20 10.1016/j.healthpol.2005.07.002 16112771

[B14] Yang Liu The current situation of commercial health insurance in China and development strategies Health Economics Research2004a 06 23 25

[B15] Bloom G Xingyuan G Health sector reform: Lessons from China Social Science and Medicine 1997 45 351 360 10.1016/S0277-9536(96)00350-4 9232730

[B16] Gui-jie Wang Shi-jun Lin Xue-mei Iu Investigation of Medical Practitioners' Perception of Safeguarding Patients' Rights Medicine and Philosophy 2005 26 6 79 80

[B17] Ministry of Health and Social Affairs The Swedish Health and Medical Services Act http://www.regeringen.se/content/1/c6/02/31/25/a7ea8ee1.pdf

[B18] Tonggan Zhao Health Legislation 2001 Beijing: People's Medical Publishing House 9

[B19] Gui-jie Wang Shi-jun Lin Xue-mei Iu Investigation of Medical Practitioners' Perception of Safeguarding Patients' Rights Medicine and Philosophy 2005 26 6 79 80

[B20] Institute Swedish The Health Care in Sweden http://www.sweden.se/upload/Sweden_se/english/factsheets/SI/SI_FS_10_Health%20care%20in%20Sweden/SI_healthcare_lowres.pdf Fact Sheet

[B21] Youlong Gong Social Medicine 2000 Beijing: People's Medical Publishing House 8

[B22] Han-jun Song Si-zheng Jiang Jia-bin Luo Yan-feng Liang The exploration and practice of a medical education model and objectives in the new situation Chinese Medicine Education Magazine 2005 26 3 7 9

[B23] Brogren PO Saltman RB Building primary health care systems: A case study from Sweden Health Policy 1985 5 313 329 10.1016/0168-8510(85)90049-1 10275594

[B24] Hjelm K Nyberg P Apelqvist J Chronic lower leg ulcers in Sweden - A survey of wound management in Primary health care, nursing homes and home care Journal of Wound Care 2000 3 133 38 10.12968/jowc.2000.9.3.25968 11933295

[B25] Blomqvist P The Choice Revolution: Privatization of Swedish Welfare Services in the 1990s Social Policy & Administration 2004 38 139 155

[B26] Skinner MW Joseph AE Kuhn RG Social and environmental regulation in rural China: bringing the changing role of local government into focus Geoforum 2003 34 267 81 10.1016/S0016-7185(02)00077-5

[B27] Chan YL Reform in t5he governance and responsibility management systems in Chinas hospital sector CMA Management 2009 31 37

[B28] Liang Wangnia Health care management 2003 Beijing: People's Medical Publishing House 7

[B29] Hu s Tang S Liu Y Escobar M De Ferranti D Health System Reform in China 6. Reform of how health care is paid for in China: challenges and opportunities Lancet 2008 372 1846 52 10.1016/S0140-6736(08)61368-9 18930520

[B30] Ke-fu Hu The Formation and Development of the New China's Socialist Healthcare and Disease Prevention System Contemporary China History Studies 2003 05 35 37

[B31] Yu Jun Lu-lu Zhang Functional problems of Chinese disease control and prevention institutions Acad J Sec Mil Med Univ 2005 11 1230 1232

[B32] Lian-Sheng Zhang Yan-Xia Zhao Cui Dan Development actuality and suggestion of maternal and child health service of communities in China Maternal and Child Health Care of China2006b 12 13 15

[B33] Swedish Tax Agency Taxes in Sweden. A summary of the Tax Statistical Yearbook of Sweden 2009 http://www.skatteverket.se/blanketterbroschyrer/broschyr/info/104.4.39f16f103821c58f680007193.html

[B34] Försäkringskassan Social Insurance in Sweden http://www.forsakringskassan.se/sprak/eng/in_brief_about_social_insurance_(for_those_who_have_recently_arrived_in_sweden)

[B35] Socialdepartementet No easy choices: The difficult priorities of health care. Report by the Health Care and Medical Priorities Commission 1993 Stockholm: Regeringskansliets offsetcentral

[B36] Youlong Gong Designing environmental health objectives in the comfortable society Primary Health Care 2003 6 171 173

[B37] Song-quan Shen The reform and sustainable development of medical insurance system Chinese Health Resources 2005 8 1 27 29

[B38] Jia Yan Zhen-quan Zheng Jin-song Yang Comparison between Domestic and Overseas Medicaid Strategies for Poor Citizens Journal of Fujian Medical University 2006 02 25 29 (Social Science Edition)

[B39] Bergman R Saltman SE Renovating the Commons: Swedish Health Care Reforms in Perspective Journal of Health Politics, Policies and Law 2005 30 253 275 10.1215/03616878-30-1-2-253 15943396

[B40] Chong-yang Ou Lu-lu Zhang Zu-xing Yang Ling Li Yang Lu Systematic dynamics model of health resources input and utilization in China Acad J Sec Mil Med Univ 2005 26 1 1220 1223

[B41] Lu-lu Zhang Shan-lian Hu Ying Wei Ming Wu Analysis of the need and demand of medical services of people in the urban and suburban areas of a region Chinese Health Economics 2002 21 03 32 35

[B42] Lu-lu Zhang Yuan-feng Qiu Ling Li Chong-yan Ou Lu Yan Transformation and Thoughts on the New Two-Level Urban Health Service System Chinese General Practice2006a 9 17 1395 1397

[B43] Le-xuan Luo Yao Lan Yuan-qing Chen Comparison of Urban Community Health Service Institutions and Hospitals in the Efficiency of Health Resources Utilization in China Chinese Hospital Management 2005 25 6 63 64

[B44] Zu-xing Yang Lu-lu Zhang Ling Li Chang-yong Xie Yu-qin Ma Construction of a macro-model of urban medical delivery system Acad J Sec Mil Med Univ 2005 26 11 1217 1219

[B45] Liu Jun Review and analysis of health sector reform in the last decade in Shanghai: An introduction of situation analysis for health policy in Shanghai Chinese Health Resources 2003 6 4 152 154

[B46] Xiao-hua Ying Guo-hong Li Shan-lian Hu Research on equality in the financial contributions of households to the health system Chinese Hospital Administration 2004 8 35 38

